# Depletion of nuclear histone H2A variants is associated with chronic DNA damage signaling upon drug-evoked senescence of human somatic cells

**DOI:** 10.18632/aging.100507

**Published:** 2012-12-06

**Authors:** Mary F. Lopez, James Tollervey, Bryan Krastins, Alejandra Garces, David Sarracino, Amol Prakash, Maryann Vogelsang, Glenn Geesman, Augusto Valderrama, I. King Jordan, Victoria V. Lunyak

**Affiliations:** ^1^ BRIMS, Thermo Fisher Scientific, Cambridge, MA; ^2^ Buck Institute for Research on Aging, Novato, CA; ^3^ Georgia Institute of Technology, Atlanta, GA; ^4^ University de Libra, Center of Excellency Regenerar, Cali, Colombia

**Keywords:** γH2A.X, DNA damage, senescence, LS-MS analysis, quantitative proteomic, SRM, histone H2A family, chromatin, DNA repair, HCA2 primary fibroblasts, epigenetics

## Abstract

Cellular senescence is associated with global chromatin changes, altered gene expression, and activation of chronic DNA damage signaling. These events ultimately lead to morphological and physiological transformations in primary cells. In this study, we show that chronic DNA damage signals caused by genotoxic stress impact the expression of histones H2A family members and lead to their depletion in the nuclei of senescent human fibroblasts. Our data reinforce the hypothesis that progressive chromatin destabilization may lead to the loss of epigenetic information and impaired cellular function associated with chronic DNA damage upon drug-evoked senescence. We propose that changes in the histone biosynthesis and chromatin assembly may directly contribute to cellular aging. In addition, we also outline the method that allows for quantitative and unbiased measurement of these changes.

## INTRODUCTION

Functional differences between the cells in an organism are defined by epigenetic factors and epigenetic programs, which are critical for the preservation of functional integrity of the cellular phenotypes [1-3]. With aging, such “epigenetic memory” of the cellular identity goes awry, contributing to the deterioration of the specificity of transcriptional programs and fidelity of genome maintenance [4-6]. These events have a devastating consequence on tissue and organ homeostasis at both the cellular and organismal level [6, 7]. Indeed, several lines of evidence indicate that with aging, chromatin undergoes major structural and functional rearrangements, but the exact cause and consequences of these changes are not yet fully understood [8-11].

Chromatin is viewed as an operational interface for almost all known nuclear processes [12, 13]. Nucleosomal packaging and histone modifications dictate the different degrees of primary chromatin compaction [14] achieved by additional chromatin structural proteins. For example, euchromatic chromatin fibers contain six nucleosomes per 11 nm; however, heterochromatin consists of 12–15 nucleosomes per 11 nm [12]. A dynamic balance between these two radically different chromatin compaction states is at the very core of the high-level nuclear chromatin organization (nuclear architecture), and is vital for maintaining cell-type identity over time [15-18].

Despite an ongoing debate regarding a cause and consequence in the hierarchy of epigenetic mishaps during cellular aging, it is becoming evident that the aging cells experience a global loss or spatial and temporal redistribution of the heterochromatin [4, 8, 1]. This phenomenon, supported by numerous studies ([20] and reviewed in [11]), may be a causative factor of senescence-associated chronic DNA damage response (DDR) [21], telomere's shortening [22, 23], de-repression of retrotransposons [24], pathologies associated with the Hutchinson-Gilford Progeria Syndrome (HGPS) [25-27], as well as altered aging cellular metabolism [28]. Recent findings demonstrate that maintenance of the heterochromatin may prolong longevity in a variety of organismal aging models, from yeast to humans [19, 28, 29].

Changes in chromatin compaction (euchromatin vs. heterochromatin) can be brought about in various ways, including histone variant incorporation or chemical post-translational modification of amino acids in the histone core and tail domains (histone PTMs) [3, 14]. These observations are reinforced by the discovery of aging-related deviations in the enzymatic machineries involved in the deposition or removal of K27me^3^H3 and K9me^3^H3 histone PTMs, which are critical for the establishment and maintenance of both constitutive and facultative heterochromatin. Although these modifications do not directly alter the structure of the nucleosome themselves, they do serve as the “landing pads” for protein complexes assembly, and are recognized by specific binding domains in a variety of non-histone proteins. These protein complexes, in turn, can exert functional effects. Biochemical or genetic manipulation of enzymatic “writers” and “erasers” of these PTMs has recently been shown to modulate germline immortality (LSD1)[30] and longevity (ASH1[31] and UTX-1[28, 29]). This suggests the importance of the euchromatin/heterochromain balance and chromatin compaction in aging.

However, as a mediator of the external signals, chromatin is anything but static. Nucleosome unwrapping and disassembly events, which must occur during DNA replication, transcription, and DNA repair, can directly influence the state of chromatin compaction [32]. Recent data also point to another pathway, other than covalent histone modification, which can profoundly alter a chromatin arrangement in aging cells. An elegant study in yeast has demonstrated that replicative aging is accompanied by a profound loss of histone proteins from chromatin, suggesting that histone deficiency *per se* can directly contribute to the changes in nucleosome density, and could ultimately lead to the loss of appropriate chromatin compaction [33]. Furthermore, the same group has established that a significant lifespan extension can be achieved by ectopic overexpression of histones in wild-type yeast. This pro-longevity pathway is distinct from other known lifespan extension pathways [9,33].

Currently, the mechanisms that underpin this profound histone loss with aging are not well understood. It is plausible, however, that this phenomenon reflects a chromatin dynamic that is intimately linked to the particularities of the DDR in the aging systems. In fact, several lines of evidence obtained in *Drosophila*, yeast, and plants indicate that a chromatin undergoes disassembly during the onset of DNA double-strand breaks (DSB) repair at the DSB sites. Importantly, the recovery from DNA damage checkpoints requires restoration of chromatin structure, which depends on re-deposition of the histones back onto the DNA by means of coordinated action of histone-chaperones and ATP-dependent chromatin remodeling complexes (reviewed in [34]). Therefore, if this “access, repair, restore” model of the chromatin dynamics is critical for the successful DNA repair and cell cycle control, then it is plausible that misregulation or interruption of the DNA damage-specific chromatin disassembly/reassembly pipeline could potentiate cellular senescence. One way to gain insight into this hypothesis is to drive the development of quantitative methods for measurements of nuclear histone composition dynamics in response to DNA damage and upon cellular senescence.

In the present study, we set out to quantify the changes in the chromatin composition in primary human fibroblasts HCA2 upon acute DNA damage and during drug-evoked senescence. By focusing on the histone H2A family members, we describe the quantitative, unbiased, and direct measurement of nuclear abundance of these components of nucleosomal organization by multiplexed selected reaction monitoring (SRM) analysis [35]. Consistent with the reduction in histone proteins upon replicative aging in yeast [33]and lowered histone H3 as well as H4 biosynthesis upon chronic exposure to DNA damage signals in human somatic cells [36], our data indicate a striking decrease in nuclear protein levels of histone H2A variants upon drug-evoked senescence in human primary fibroblasts. These results further reinforce the evidence that histones deficiency in the chromatin following senescence-associated chronic DDR might impose dramatically different chromatin condensation state than the one observed during normal proliferation. In addition, the current study presents experimental evidence that DNA-damage can be accurately and efficiently measured by scoring H2A.X phosphorylation events (γH2A.X) in SRM experiments. By providing an excellent sensitivity, specificity, and precision, the method described in this study represents an alternative for quantitative histones and virtually any histone PTM measurements, which is far superior to immunofluorescence microscopy and western blot analysis.

## RESULTS

### Modeling of acute DNA damage and genotoxic stress (drug-evoked) senescence in human somatic HCA2 cells

Senescence is triggered by various cellular stresses that result in genomic lesions and activation of chronic DNA damage signaling. However, the role of chromatin and its properties upon induction of senescence remains highly controversial [37-41, 83]. Several chemo-therapeutic drugs, including etoposide [41, 42], bleomycin [36, 43], and doxorubicin [43] are known to induce premature senescence in a variety of human somatic and stem cells. In our study, we have used well-characterized model of human neonatal fibroblasts (HCA2) [44-46] to assess the nuclear H2A histone levels under three different conditions: 1) normally proliferating cells, 2) acute DNA damage, and 3) drug-evoked senescence.

**Figure 1:**
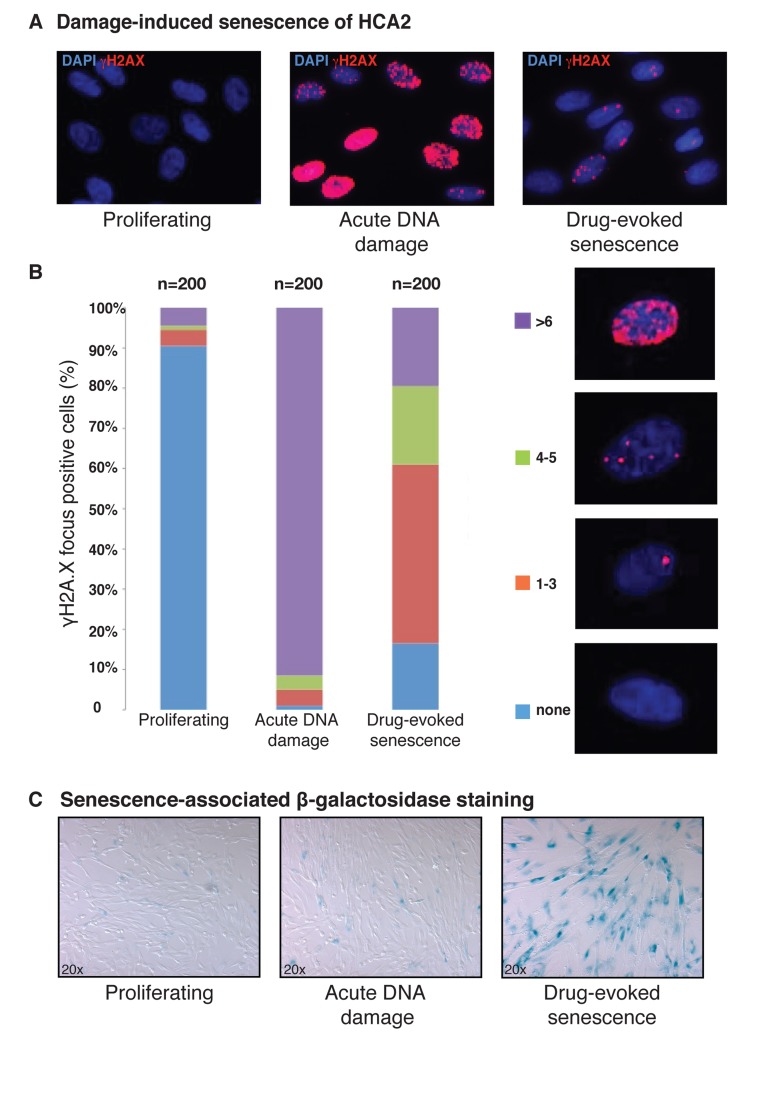
Characterization of drug-evoked senescence of HCA2 fibroblasts (**A**) HCA2 cultures stained with anti γH2A.X. Blue, DAPI; red, γH2A.X. Each image represents a projection of all optical sections through a cellular culture. Representative images are shown for each condition: Normal proliferating HCA2 cells (−Bleo), same cells under conditions of acute DNA damage (+Bleo, 3 h), and drug-evoked senescence (+Bleo, 5 days). (**B**) Quantification of γH2A.X-foci frequency upon bleomycin treatments as described earlier. Typical representations of cells with varying numbers of γH2A.X foci per cell are shown in the right column. On an average, more than 200 cells were screened per condition in independent experiments. (**C**) Staining for the marker of cellular senescence, senescence-associated β-galactosidase. Drug-evoked senescence HCA2 cells (+Bleo, 5 days) exhibited both an increase in senescence-associated β-galactosidase levels and morphological alterations.

Cycling early passage diploid HCA2 fibroblasts were cultivated as outlined in *Materials and Methods* Section, and treated with the chemomimicking agent, bleomycin (50 μg/ml), for 3 h to generate conditions of acute DNA damage. Drug-evoked senescence was induced when the cells were cultured in normal growth media after the removal of bleomycin for 3-5 days [36, 44]. A functional biomarker that is used most often to detect activated DDR is the histone variant, H2A.X, which when phosphorylated by ATM/ATR on serine 139 is commonly known as γH2A.X [47, 48] (Figure 1A). Under the condition of acute DNA damage, about 90% of the cells were positive for defused global nuclear staining in immunostaining experiments with γH2A.X-specific antibodies (Figure 1B). Despite the initial wave of DNA repair, leading to progressive reduction in time of the bleomycin-induced γH2A.X foci, about 80% of the cells underwent drug-evoked cellular senescence after 3-5 days post-bleomycin treatment. This was indicated by the formation of the predominantly focal pattern of gH2A.X and activation of lysosomal senescence-associated b-actin (Figure 1C), which is in accordance with the previously reported observations in the same cell system [49]. These events are known to result in constitutive DNA damage signaling, which is a phenomenon well documented in all major types of senescence [23, 50, 83].

**Figure 2:**
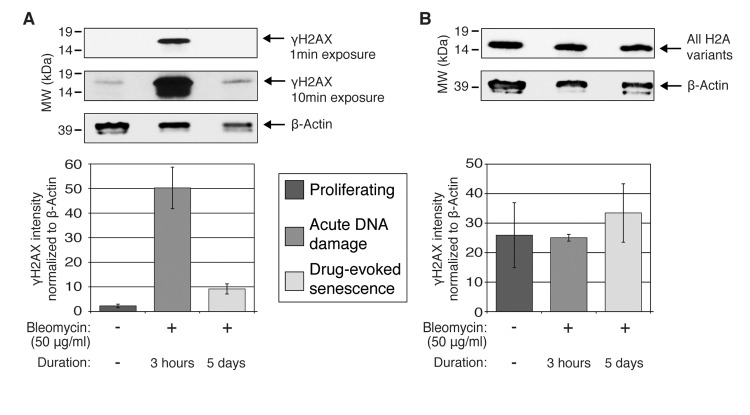
Quantification of γH2A.X and total H2A variants by western blot Whole-cell extracts were prepared from untreated proliferating and bleomycin-treated HCA2 cells (acute DDR (+Bleo, 3 h) and drug-evoked senescence (+Bleo, 5 days)), and subjected to western blot analysis with (**A**) γH2A.X and (**B**) generic H2A antibodies. Two exposures (short: 1 min and long: 10 min) are shown for the γH2A.X staining. β-actin was used for normalization purposes. Graphs represent computationally calculated band intensities for each antibody, normalized to β-actin, from western blots repeated for biological triplicates of each condition.

Western analysis of HCA2 cells upon proliferation, acute DNA damage, and drug-evoked senescence revealed a significant increase in γH2A.X representation in the whole cell extracts during acute DNA damage (Figure 2A), and only a marginal increase in γH2A.X upon drug-evoked senescence, when compared with the proliferating cells. However, no significant change in the total protein level of histone H2A was observed by western blot analysis in the tested samples (Figure 2B). Most antibody-based tests cannot provide the requisite specificity for the accurate quantification of proteins in question, because multiple isoform or variants of the same protein could be recognized by the same antibody. The data also demonstrate that different exposures in western blot analysis can significantly impair interpretations (Figure 2A, compare upper and middle panels). The antibodies used are listed in *Materials and Methods* Section.

### Development of multiplexed SRM assay for quantitative measurement of the histone H2A family, H2A.X and γH2A.X

Over the past few years, mass spectrometry has emerged as a technology to complement and potentially replace standard immunoassays in routine research and clinical tests. Mass spectrometry based SRM is rapidly becoming a preferred technology for the development of quantitative protein or peptide assays for clinical and academic research because it delivers high sensitivity, selectivity, throughput, and accurate quantification [35]. In an SRM experiment, the two mass analyzers of a triple-quadrupole mass spectrometer are used to isolate the peptide ion of interest and the derived fragment ion(s). This enables the precise quantification of peptides in a complex biological background, and thus indirectly the corresponding proteins present in the samples. The ability to multiplex the analytes allows effective quantification of protein panels across multiple samples by monitoring specific transitions (precursor-fragment ion pairs) during a predefined elution window. Targeted proteomics approaches based on SRM coupled to high-resolution LS-MS/MS discovery workflows have proven effective for the analysis of biomarkers in plasma and serum, successfully permitting a systematic, quantitative analysis of putative marker candidates [51, 52].

In this study, we developed a multiplexed SRM assay for quantitative measurement of: 1) nuclear representa-tion of histone H2A family proteins (histone H2A variant unbiased approach), 2) abundance of histone H2A.X (variant-specific approach), and 3) histone H2A.X phosphorylation (γH2A.X) (PTM-specific approach). Peptides selection was based on three different criteria: literature references, previously obtained LC-MS/MS discovery data (not shown), and algorithmic prediction. An initial exhaustive list of peptides and transitions was iteratively optimized as previously described [52-54]. The peptides sequences and transitions for monitoring peptides and heavy standards are listed in Table 1 and Supporting Information Table S1. Calibration curves with limit of detection (LOD) and limit of quantification (LOQ) for the peptides are shown in Supporting Information Figure S1.

**Table 1:** Histone H2A.X Peptide sequences, abundance ratios, and %CVs

Peptide Sequence	Peptide Type	Description	Parent Mass	Ratio of Sample Groups (-Bleo : +Bleo 3 hrs : +Bleo 5 days)	Internal Standard Mass	Proliferating (-Bleo)		Acute DNA damage (+Bleo 3 hrs)		Drug-evoked senescence (+Bleo 5 days)	
						Mean Calculated Amount (fmol)	CV%	Mean Calculated Amount (fmol)	CV%	Mean Calculated Amount (fmol)	CV%
AGLQFPVGR	Wild type	All H2A variant monitoring peptide	472.769	1.0 : 1.4 : 0.46	517.255	101487.7438	8	139691.2925	3	38081.93125	3
HIQLAIR	Wild type	All H2A variant monitoring peptide	425.766	1.0 : 1.4 : 0.55	517.255	24286.20625	8	32711.15375	4	10889.4625	3
TSATVGPK	Wild type	H2A.X specific monitoring peptide	380.713	1.0 : 1.4 : 0.74	517.255	234.1725	11	310.36	5	140.9125	5
ATQASQEY	Wild type	C-term serine, fully tryptic	897.394	1.0 : 0.8 : 0.51	901.401	203.7625	15	192.395	7	115.4125	13
A*TQASQEY	Heavy standard	C-term serine, fully tryptic	901.401	1.0 : 1.2 : 1.1	None	137.22	11	161.0325	10	151.74375	11
ATQAS*QEY	phosphorylated	C-term serine, fully tryptic	489.184	1.0 : 4.0 : 2.3	491.187	0.745	49	2.965	16	1.345	51
A*TQAS*QEY	Heavy phosphorylated standard	C-term serine, fully tryptic	491.187	1.0 : 0.98 : 0.78	None	163.08375	6	159.91	6	127.00375	9
KATQASQEY	Wild type	C-term serine, missed cleavage	513.248	1.0 : 0.87 : 0.48	517.255	26.85625	11	22.06	11	10.31	16
K*ATQASQEY	Heavy standard	C-term serine, missed cleavage	517.255	1.0 : 0.95 : 0.81	None	163.1425	6	154.51	4	132.3475	5
KATQAS*QEY	phosphorylated	C-term serine, missed cleavage	553.231	1.0 : 2.4 : 1.3	557.238	0.945	45	2.255	38	0.985	54
K*ATQAS*QEY	Heavy phosphorylated standard	C-term serine, missed cleavage	557.238	1.0 : 1.0 : 0.81	None	159.695	6	160.75875	6	129.5475	9

**Figure 3:**
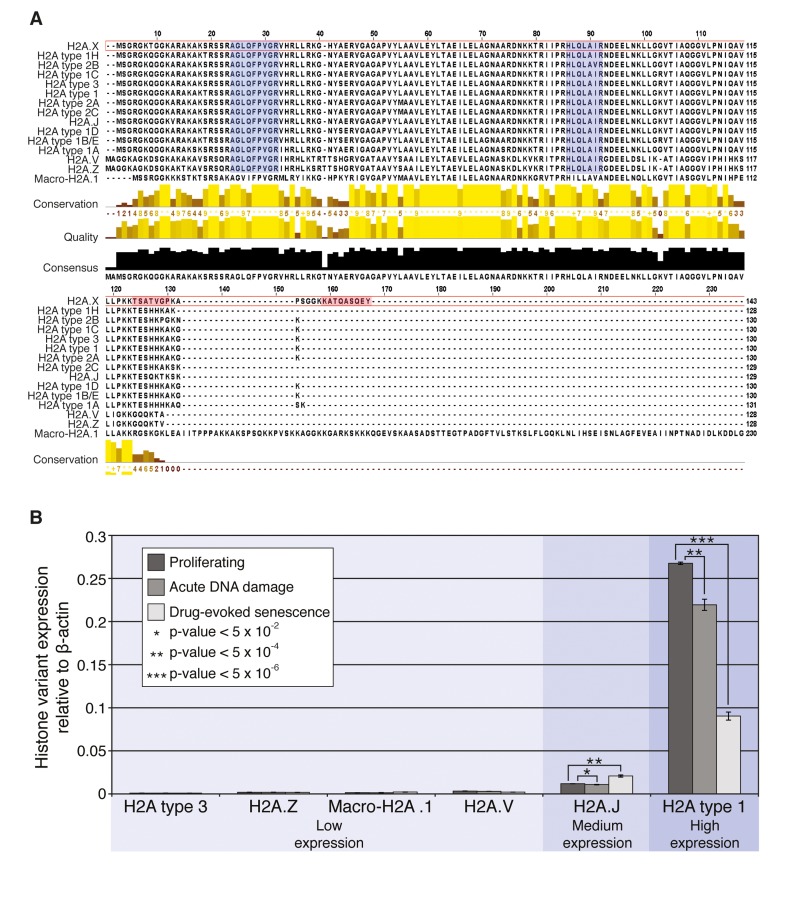
Histone H2A variants and their transcription in HCA2 cells (**A**) Alignment of all H2A variant protein sequences. Quality track scores the level of sequence conservation between all variants for each amino acid. Blue boxes highlight tryptic histone H2A family internal monitoring peptides used in SRM analysis. Red boxes highlight tryptic H2A.X-specific peptides. (**B**) RNA was prepared from untreated proliferating and bleomycin-treated HCA2 cells (acute DDR (+Bleo, 3 h) and drug-evoked senescence (+Bleo, 5 days)). RT-PCR analysis was used to quantify the mRNA abundance of a selection of H2A variants in each condition. H2A type 1 represents a number of H2A variants from the type 1 cluster (see **A**). The *p*-values for *H2A.J* and *H2A type 1* are shown, calculated using Student's *t*-test, two-tailed, unequal variance, between the conditions. Each condition consists of biological triplicates, with further technical triplicates for each. Histone H2A family members can be subdivided into low expression (*H2A type 3, H2A.Z, macro-H2A.1, H2A.V*), medium expression (*H2A.J*), and high expression (*H2A type 1*), based on their expression relative to *β-actin*.

In the *unbiased approach*, the internal monitoring peptides AGLQFPVGR and HLQLAIR are non-unique and common to all of the H2A histone family members (Figure 3A) with the exception of macro-H2A histone. These peptides allowed quantification of the abundance of all H2A variants in the tested samples. In the *variant-specific approach*, the other internal monitoring peptide, TSATVGPK, allowed for the specific quantification of H2A.X histone, regardless of its phosphorylation status. The C-terminal peptides ATQAS_139_QEY and KATQAS_139_QEY (missed cleavage), both phosphorylated and unmodified, allowed for a quantitative measurement of the serine 139 phosphorylated and unmodified tryptic products unique to histone H2A.X in our *PTM-specific approach* (Figures 3A and 5A). Simultaneous quantitative measurement of all of these tryptic peptides allowed us to monitor overall nuclear representation of histone H2A family members, H2A.X, and, importantly, allowed us to estimate the rate of phosphorylation of H2A.X histone by monitoring γH2A.X peptide in the tested samples.

### Expression of H2A histone family members is sensitive to acute and chronic DNA damage signaling

Having established that all, except macro-H2A.1, histone H2A family members are highly conservative in their globular domain at the level of their protein sequence, we subsequently investigated the genomic sequence similarities among the histone H2A family members (shown in Supporting Information Figure S2). Histone variant specific primers were designed wherever possible using NCBI Primer-BLAST, and verified on the UCSC genome browser (Supporting Information Figure S3). Not all variants yielded unique primer pairs. The qPCR analysis was performed as described in *Materials and Methods* Section to investigate the dynamics of the variants-specific expression in normally proliferating HCA2 cells as well as in the same cells under condition of acute DNA damage and drug-evoked senescence. The results are shown in Figures 3B and 5B. Using gene-specific single-stranded DNA preparation coupled with qPCR, we were able to selectively assess the transcriptional activities of seven genes of H2A histone variants family: *H2A type 3*, *H2A type 1, H2A.Z, H2A.V, H2A.J, H2A.X*, and *marco-H2A.1*. We noted that the overall level of transcriptional activity of these genes varied, which can be arbitrary sub-divided into three distinct categories, where only *H2A type 1* genesexhibited high-level expression. This cluster of histone variants probably accounts for the majority of histone H2A protein within the chromatin of HCA2 cells (Figure 3B). Both histones *H2A.J* (Figure 3B) and *H2A.X* (Figure 5B) exhibited medium levels of gene expression with only marginal differences in the amounts of mRNA generated from these genes, when compared with *β-actin* gene expression. *H2A type 3*, *H2A.Z, H2A.V*, and *marco-H2A.1* expression was barely detectable in HCA2 fibroblasts. Thus, we rationalized that histone *H2A type 1*, *H2A.J*, and *H2A.X* are the major contributors to the chromatin packaging in HCA2 cells.

Having established the major transcribed members of the histone H2A family, we subsequently examined the changes in transcriptional activities of these genes upon acute and chronic DNA damage signaling. It has been previously demonstrated that biosynthesis of histones H3 and H4 drastically declined in the tested somatic models of *ex-vivo* replicative and drug-evoked senescence, IMR90 and WI38 [36]. In accordance with these observations, our data demonstrated that acute and chronic DNA damage signaling also impacts the expression of histones *H2A type 1* and *H2A.X*, resulting in statistically significant (*p-*values=3.85×10^−7^ and 1.22×10^−5^, respectively*)* downregulation of these histones at the transcriptional level (Figures 3B and 5B) under both acute DNA damage and drug-evoked senescence conditions. Therefore, we concluded that expression of these histones is sensitive to DNA damage signaling and activation of chronic DDR, in particular. However, no changes in the transcriptional activity of *H2A type 3*, *H2A.Z, H2A.V, H2A.J*, or *marco-H2A.1* genes were observed under the same conditions.

The above-mentioned data further expand the previous observation of changes in the histone H3 and H4 transcription during *ex-vivo* replicative aging and drug-evoked senescence of fibroblasts [36], and demonstrate that similar trends can be recorded for another indispensible nucleosomal packaging histone family, H2A. Comparison of the expression levels of the different members of the histone H2A family revealed that not all of the histone family members respond equally to the chronic DNA damage signaling. Particularly, histone *H2A.J* transcription showed slight, but significant transcriptional upregulation.

### Drug-evoked senescence is associated with significant depletion of the histone H2A variants in the chromatin of human primary fibroblasts

To biochemically quantify the histone H2A variants' protein levels in the nucleus of cells subjected to either acute or chronic DNA damage signaling, we deployed SRM, which allowed us to monitor the abundance of H2A type 1, H2A.X, and H2A.J gene products at the protein level. The optimized SRM assay was used to interrogate nuclear fraction samples isolated from proliferating (−bleomycin) HCA2 cells, as well as from the cells under condition of acute DNA damage (+bleomycin, 3 h) and drug-induced senescence (+bleomycin, 5 days). The nuclei fractions were prepared as described in the *Materials and Methods* Section. Eight independent experimental samples were obtained for each condition. All the samples were normalized by DNA content. Quantitative data were obtained for all samples and then analyzed in “*Pinpoint”* software to determine the differential abundance patterns of all peptides described in the previous section (Table 1).

**Figure 4:**
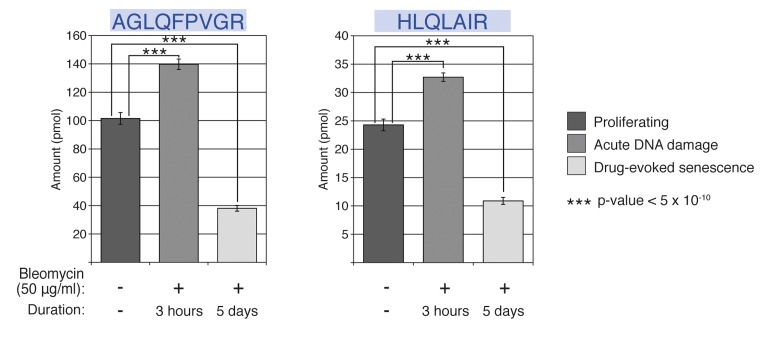
Relative abundance of histone H2A family in proliferating and bleomycin-treated cells The AGLQFPVGR and HLQLAIR peptide sequences are common to all H2A histone family types (**3A**). The relative nuclear abundance of both the peptides, as measured by SRM, is shown for proliferating, acute DNA damage, and drug-evoked senescence HCA2 cells. Eight biological replicates were used for each condition. The *p*-values were calculated using Student's *t*-test, two-tailed, unequal variance, between the conditions.

Our data revealed a prominent and statistically significant loss of the histone H2A family proteins in the HCA2 human fibroblasts under conditions of chronic DDR upon drug-evoked senescence (p-value= 1.22×10−15 for AGLQFPVGR monitoring peptide and p-value= 2.12×10−14 for HLQLAIR monitoring peptide) (Figure 4). This observed downregulation of H2A protein abundance in the nuclei of senescent cells correlates to the decline in the accumulation of mRNA of the HCA2-expressed histone H2A variants shown inFigure 3B, suggesting that either biosynthesis or their delivery into the nuclei was abrogated upon chronic DDR. The raw quantitative data for all sample measurements are given in Supporting Information Table S2.

Interestingly, our data also suggest a biphasic mode for nuclear dynamics of these histone H2A variants in response to drug-induced genotoxic stress. Prior to decline in the nuclear H2A protein levels in senescent cells, there was a statistically significant increase (*p*-value= 5×10^−10^) in the representation of these histones within the nuclei of the HCA2 cells during acute DDR (3 h after the addition of bleomycin to the media). We speculate that this increase in the nuclear H2A histones might be due to the formation of a histone supply “buffer zone,” which assists the active chromatin remodeling and histone exchange events coupled at this stage with efficient DNA damage repair, similar to the one described in [55]. While we observed an initial increase (about 36%) in the nuclear abundance of histone H2A family members, our data clearly depict a second trend: Histone H2A family nuclear representation dropped by 70%, when compared with the stage of acute DNA damage and 60%, when compared with the initial amount of histones H2A in normally proliferating HCA2 cells. One reason for such dynamics could be that the DNA repair machinery encountered genomic regions within which DNA damage could not be resolved efficiently, perhaps due to the failure of either site-specific or global action of the histone chaperones [34, 55, 56]. In general, histone chaperones are responsible for both the delivery of histones into the nucleus and integration of histones into chromatin, to faithfully restore the chromatin landscape following DNA repair. Irrespective of the nature of these events, it is important to emphasize that this biphasic behavior would not be detected by traditional western blot analysis of histone H2A, because these changes were not detectable in the western blot analysis experiments using total cellular HCA2 protein extracts (Figure 2B).

Together, these data support the previous observations in yeast and mammalian somatic cells, where replicative aging has been noted to be associated with prominent loss of major histones from the chromatin [33, 36]. In addition, our data also indicate that the SRM approach has a higher discriminating potential than traditional antibody-based assays.

### Measurement of histone H2A.X abundance and H2A.X phosphorylation upon acute and chronic DNA damage signaling

**Figure 5:**
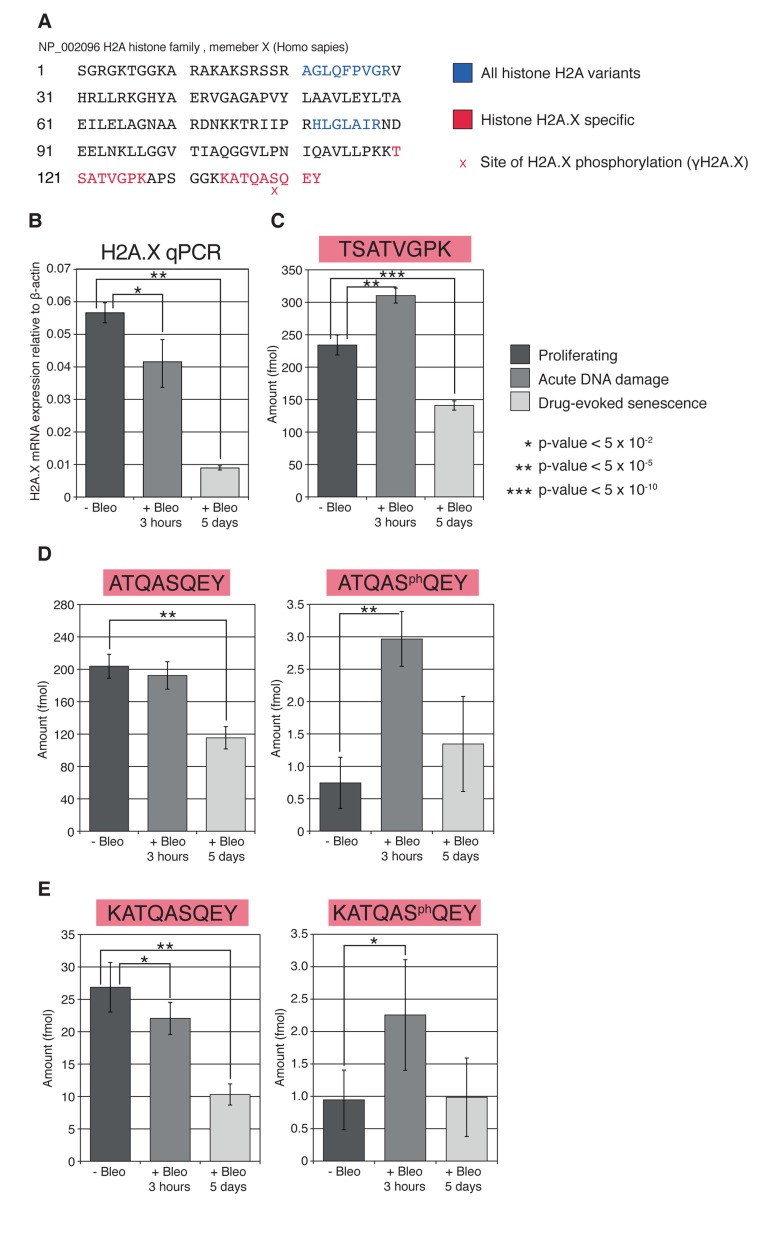
Relative abundance of unique phosphorylated, unphosphorylated C-terminal, and internal monitoring H2A.X peptides measured by SRM and qPCR (**A**) H2A.X sequence indicating tryptic peptide sequences common to all H2A family members (blue) and unique to H2A.X (red). The location of serine phosphorylation found in γH2A.X is marked. (**B**) qPCR analysis of *H2A.X* mRNA expression in proliferating, acute DNA damage, and drug-evoked senescence HCA2 cells, as described in **3B**. Biological and technical triplicates were performed for each condition. The *p*-values were calculated using Student's *t*-test, two-tailed, unequal variance. (**C**) Relative nuclear abundance of the unique internal monitoring H2A.X peptide, TSATVGPK, as measured by SRM. Eight biological replicates were used for each condition. (**D, E**). Relative nuclear abundance of the unique C-terminal phosphorylated and unphosphorylated H2A.X peptides. The fully tryptic ATQASQEY (**D**) and one missed cleavage KATQASQEY (**E**) peptides and their phospho-Ser-139 analogs (γH2A.X) were quantified using SRM. Eight biological replicates were used for each condition, for each peptide.

H2A.X is constitutively expressed throughout the cell cycle. This histone variant differs from any other core histone H2A family member by the presence of a short C-terminal tail and a highly conserved SQE motif (serine, glutamine, glutamic acid) (Figures 3A and 5A). It has been reported that the H2A.X expression levels vary between mammalian tissues, ranging from 2 to 10% of total histone H2A expression [57]. This histone variant undergoes rapid phosphorylation of serine at position 139 (site of γ-phosphorylation) by members of the PI-3 kinase-like family in response to the DSBs [55]. The phosphorylation event serves as an important cue for the stable retention of DNA damage repair and DDR mediator proteins in close proximity to DSBs, resulting in the formation of microscopically discernible foci that function as a platform for interactions between multi-component signal transduction complexes, facilitating the activation of the cell cycle checkpoint [58-60]. The formation of γH2A.X foci can occur in response to genotoxic insults, such as drug treatment, UV exposure, metabolic stress, reactive oxygen species (ROS) [55], as well as under conditions of inappropriate over-activated growth and signaling pathways [82].

While both the timing and order of DDR events preceding and following H2A.X phosphorylation are well documented in the context of acute DNA damage and senescence in human cells [50], there is a lack of direct quantitative measurements of nuclear γH2A.X. Our multiplexed SRM approach allows for variant-specific quantification of H2A.X protein and its phosphorylation (PTM-specific quantification) in the tested samples. The H2A.X-specific monitoring peptides are listed in Table 1 and shown in red in Figure 5A.

Consistent with the overall depletion in histone H2A variants' protein level in the nuclei of HCA2 cells (Figure 4) following chronic DNA damage signaling, we observed a robust decrease in protein representation (*p*-value= 3.05×10^−10^*)* of H2A.X histone variant (Figure 5C). This was also accompanied by statistically significant decrease in H2A.X at transcriptional level (*p*-value= 1.22×10^−5^*)*, as demonstrated by qPCR analysis (Figure 5B).

Interestingly, our measurements indicate that histone H2A.X comprises only about 0.4% of the total H2A protein in the nuclei of normally proliferating HCA2 cells (124 *pmole* of total H2A family member proteins shown in Figure 4 vs. 450 *fmole* of total H2A.X protein presented in Figure 5). This nuclear abundance is 10-fold lower than that previously suggested in mammalian tissues based on immunostaining analysis and 2D gel electrophoresis [57]. H2A.X nuclear protein dynamics were noted to follow a similar biphasic mode, as shown in Figure 4 and discussed earlier. We observed a relatively small, but statistically significant increase (~36%, *p*-value= ~2.20×10^−11^) in the amount of nuclear H2A.X in the condition of acute DNA damage.

The reproducibility of SRM quantification is dependent on the efficiency of trypsin digestion. Experimental measurements may be skewed if protein modifications block the expected proteolytic cleavage site due perturbations in the protein folding. Therefore, in addition to fully tryptic peptides, we also monitored the missed cleavage peptides KATQASQEY and KATQASphQEY, because these were detected in our discovery spectra. The relative amounts of the fully tryptic and missed cleavage peptides in our experiments indicated that although about 10% of the unphosphorylated protein in all of our samples had one missed cleavage, the relative amounts of semi-tryptic KATQASQEY across all three samples were similar to the fully tryptic peptide ATQASQEY (Figure 5D and 5E, Table 1). Similar results were obtained with the phosphorylated peptides ATQASphQEY and KATQASphQEY (Figure 5D and 5E, Table 1).

As expected, SRM-targeted analysis of the H2A.X-specific tryptic peptides encompassing the serine 139 site for the damage-induced phosphorylation, ATQASQEY, and KATQASQEY revealed a decrease in the unphosphorylated form of H2A.X during acute DNA damage and in senescent cells (Figure 5D and 5E). This result coincides with the increased DNA damage-propelled serine 139 phosphorylation (γH2A.X) (Figures 1A, 1B and 2A) confirmed by quantification of ATQASphQEY and KATQASphQEY peptides (Figure 5A, 5D, and 5E). Our data indicate that the fraction of the phosphorylated H2A.X (γH2A.X) is about 0.5% relative to the total amount of H2A.X in the nucleus of the normally proliferating HCA2. Acute DNA damage has been found to result in about 3% of total H2A.X phosphorylation (comparable with that reported in [57]), where only about 2% of the total nuclear H2A.X has been noted to exist in the phosphorylated form upon drug-evoked senescence.

Our results, therefore, suggest that HCA2 fibroblasts had partially resolved bleomycin-inflicted DNA damage triggered by chronic DNA damage signaling and senescence.

Together, these data, although consistent with immunostaining and western blot analysis shown in Figures 1A, 1B, and 2A, represent the first truly quantitative measurement of the degree of H2A.X phosphorylation in human primary somatic cells under conditions of acute DNA damage and drug-evoked senescence. The observed degree of H2A.X phosphorylation in the senescent human fibroblasts is also in agreement with our previous, genome-wide nucleosomal analysis of *γ*H2A.X distribution, which estimated that only 1.2% of the total chromatin contains γH2A.X-bearing nucleosomes upon replicative senescence of human adult stem cells (ADSCs) [24].

In accordance with the nuclear loss of generic H2A (Figure 4), our measurements of total nuclear quantitates of H2A.X (both phosphorylated and unphosphorylated) indicate a nuclear depletion of this histone variant upon chronic DDR in drug-evoked model of senescence (400 *fmol* in proliferating cells vs. 260 *fmol* in senescent cells). This decline in nuclear protein abundance of histone H2A.X variant, together with robust decrease in the transcription activity of this gene (Figure 5B), suggest that chronic DNA damage signaling might be associated with downregulation of histone H2A.X biosynthesis or its trafficking from the cytoplasm to the nucleus. This observed change also corroborates with the previous reports of a decrease in the synthesis of H2A.X upon *ex-vivo* replicative aging of human primary lung fibroblasts.

## DISCUSSION

Even slight changes in the histone-DNA equilibrium disrupt DNA synthesis, compromise chromatin architecture, block cell cycle progression, and challenge cell viability [36, 56, 61]. The prominent family of histone H2A variants has recently been the focus of numerous studies concerning their role in the crucial biological processes. Variants of the histone H2A family (the largest among histone classes family) are the most diverse in sequence and exhibit distinct functions, comprising DNA damage repair, transcriptional regulation, cell cycle control, and chromatin condensation [62], [63]. The exact mechanistic aspects regarding the actions of histone H2A variant are not yet fully understood. In general, the carboxy-terminal tails of the members of the H2A histone family have been shown to contact the “linker” region of the nucleosomal DNA and histone H1 [64,65], and the members of this family can differentially affect the condensation of the chromatin fiber and its stability [65]. It is therefore reasonable to assume that this family of histones might play important roles in chromatin organization during cellular and organismal aging.

The significant reduction of H2A family protein representation, including H2A.X histone, which we observed in the nuclei of senescent primary human fibroblasts, may reflect changes in the overall structure of the senescent chromatin as well as its accessibility. Decrease in H2A protein abundance within the nuclei upon senescence is indicative of lesser nucleosomal density in the senescent chromatin, which may ultimately result in perturbed nucleosomal phasing and mobility. Considering that the re-establishment of proper chromatin structure is an essential part of the completion of DSB repair, it is highly plausible that deficiency in the nuclear histone composition may be an integral part of the chain of molecular events leading to persistent DDR and physiological changes observed in senescent cells.

One explanation for these intriguing observations relates to the possible role of histone chaperones in the mechanistic aspects of chromatin packaging during chronic DDR. Histone chaperones are proteins that can assemble histones and DNA into the nucleosome structure as well as disassemble an intact nucleosome into its subcomponents to facilitate transcription, DNA replication, and DNA repair [34]. They also function to provide guidance for histone folding, prevent histone aggregation, and mediate histone transport between the nucleus and cytoplasm [32,66, 67]. DNA repair is tightly linked to chromatin assembly, and requires the function of histone chaperones [68-70]. For instance, in yeast cells, nearly all the soluble pool of the DNA checkpoint protein Rad53 is bound to Asf1 (a histone H3/H4 chaperone) in the absence of DNA damage or replication stress. This interaction between Rad53 and Asf1 appears to prevent Asf1 from binding to histones and assembling chromatin [71,72]. DNA damage or replicative stress induced phosphorylation of Rad53 favors the release of Asf1 in the vicinity of DNA lesions, Asf1 histone binding, and promotes Asf1 chromatin assembly capacity. In human cells, even a marginal increase in histones H3 and H4 impedes the function of Asf1 and prevents chromatin disassembly, indicating that changes in the level of histones can directly influence the properties of proteins involved in their biogenesis [36, 55, 73]. It is tempting to speculate that deviated function of H2A/H2B-specific chaperon(s) might be involved in the observed phenomenon of depletion of H2A family in the chromatin in senescent fibroblasts. There are several H2A/H2B-specific chaperones, which have been reported to contribute to chromatin dynamics. These include nucleolin [74], PP2Cy [75], FACT [76], Nap-1 [77], NASP [78], and those reported in [66]. However, the molecular link between their function and chromatin reconfiguration in senescence has not yet been explored.

Interestingly, loss of nuclear histone H2A is not a direct result of DNA damage *per se*. Our data indicate a transient increase in the abundance of nuclear histone H2A family members immediately after DNA damage (acute DNA damage). This increase is not associated with transcriptional upregulation of the expressed histone H2A members, and probably reflects the formation of a nuclear “histone buffer” zone, a pool of histones that might serve to modulate two opposing activities. One purpose would be to provide for chromatin recovery from DNA damage: This histone pool might be utilized in *chromatinre-assembly* and histone replacement, concomitant with the DNA repair process. On the other hand, another purpose of histones increase in the nuclei upon acute damage might be to directly influence the properties of the proteins involved in chromatin remodeling to either block or balance the rate of *chromatindisassembly*, as supported by several lines of evidence, which indicate that even a marginal increase in histone levels impedes nuclear histone chaperones function (as exemplified in Asf1 studies [73]) and prevents chromatin disassembly.

It is important to note that chronic DDR in our model system is not a result of DNA damage accumulation, because we observed less γH2A.X upon senescence than immediately following acute DNA damage. However, we believe that the senescent HCA2 phenotype might be a result of drastic chromatin relaxation, as suggested by the quantitative measurements presented in this study and previously published data on total cellular histone H3 and H4 abundance [36]. We propose that this chromatin relaxation and histone deficiency prevents proper nucleosomal reassembly at the last stage of DNA damage repair, thus keeping DNA damage signaling armed in the senescent cells.

Recent structural, biophysical, and biochemical studies have established that both core histone chaperones and ATP-utilizing motor proteins incorporated into histone remodeling complexes can interact with DNA repair factors, and also transmit signals to the cell cycle control machinery [32]. Our data also suggest that detailed studies on histone chaperones responsible for H2A deposition and chromatin remodeling complexes that operate on the recognition of H2A-specific PTMs may shed additional light on the complex mechanisms of cellular senescence. Future studies should reveal how chromatin communicates with the chronic DNA damage signaling pathways in senescent cells.

## MATERIALS AND METHODS

### Samples

HCA2 cells were cultured in the presence and absence of 50 μg/ml of bleomycin as previously published [49, 79]. The nuclei were harvested by centrifugation through a 30% sucrose cushion as described in [80]. This resulted in three sample groups: 1. No DNA damage, 2. Early DNA damage, 3 h after bleomycin treatment, and 3. Drug-evoked DNA damage: after initial 3 h in 50 μg/ml of bleomycin, the cell media was changed to normal growth media and the cells were further cultured for 5 days and then subjected to nuclei preparation and SRM analysis. The nuclei pellets from approximately 10M cells were lysed in 400 ml 8M Gu-HCL + DTT. The samples were subsequently enzymatically digested and processed for SRM analysis as previously published [35, 52].

### Immunohistochemistry

As described in the *Samples* Section, 1×10^4^ HCA2 cells/well in 4-well slides were treated with bleomycin, and then fixed with 4% paraformaldehyde and permeabilized with PBS and 0.5% Triton X-100. The blocking and antibody incubations (γH2A.X; Millipore #05-636) were performed in 4% normal donkey serum (NDS; Jackson Immunochemicals) in PBS. The nuclei were counterstained with 100 ng/ml of 4', 6-diamidino-2-phenylindole (DAPI; Sigma), and the slides were mounted in ProLong Gold antifade aqueous mounting medium (Invitrogen). Epifluorescence images were acquired on an Olympus BX60 fluorescence microscope with Spotfire 3.2.4 software (Diagnostics Instruments).

### Senescence-associated β-galactosidase assay

As described in the *Samples* Section, 1×10^4^ HCA2 cells/well in 4-well slides were treated with bleomycin. β-galactosidase detection was carried out using a commercial senescence detection kit (BioVision #K320-250), according to the manufacturer's recommended protocol. The slides were incubated with staining solution for 14 h prior to visualization.

### Western blot analysis

A total of 1×10^5^ HCA2 cells were cultured on 10-cm dishes and treated with bleomycin, as described in the *Samples* Section. Triplicates were performed for each condition. The cells were collected by cell scraper and lysed with lysis buffer (50 mM Tris-HCl; pH 7.4; 100 mM NaCl; 1% NP-40; 0.1% SDS; 0.5% sodium deoxycholate; Halt protease inhibitor cocktail, Thermo Scientific #87786) at 4°C. The lysates were rotated at 4°C for 45 min, and then sonicated vigorously to shear DNA. Protein concentrations were determined by Bradford assay, and normalized lysate volumes were loaded onto a NuPage 4-12% Bis-Tris gel (Novex #NP0336) in NuPage LDS sample buffer (Invitrogen #NP0007), and run at 200 V for 50 min. The proteins were transferred to nitrocellulose membrane using the XCell II blot module (Invitrogen #EI9051). The membranes were blocked with 5% milk solution for 30 min at room temperature, before being incubated with primary antibodies overnight in TBS-T at 4°C with gentle rocking. The antibodies used were as follows: γH2A.X (Millipore #05-636), Histone H2A (AbCam #AB13923), and β-actin (AbCam #AB6276). HRP-conjugated secondary antibodies were used before subsequent ECL exposure. The γH2A.X membranes were exposed for short (1 min) and long (10 min) periods, and the representative results are shown in Figure 2A. The band intensities were calculated using Fiji (Image J), and normalized to that of β-actin.

#### Quantitative PCR

### RT-PCR

A total of 1×10^5^ HCA2 cells were cultured on 10-cm dishes and treated with bleomycin as described in the *Samples* Section. The procedure was performed in triplicates for each condition. The cells were collected by cell scraper and RNA was isolated using Trizol reagent (Life Technologies #15596). DNA contamination was removed with TURBO DNase (Applied Biosystems #AM2238) at 37°C for 30 min, followed by 10 min at 70°C in the presence of 15mM EDTA to inactivate the enzyme. Reverse transcription was carried out using gene-specific primers (see the following paragraph) and SuperScript III reverse transcriptase (Invitrogen #18080-051) at 55°C for 1 h. qPCR was carried out using Fast SYBR Green Master Mix (Applied Biosystems #4385612). The reactions were run in a Roche Lightcycler 480 system under the following conditions: 1 × 95°C for 20 s; 40 × 95°C for 3 s, and 60°C for 30 s. Crossing point (Cp) cycle values for each reaction were calculated by Absolute Quantification/Second Derivative Maximum calculations. The Cp values for each primer/sample were normalized to that of β-actin expression.

### Primers

Also refer to Supplemental Figure S1. The primers were designed using NCBI Primer-Blast software using default parameters. This software was used to design primers that were specific for individual H2A variants (wherever possible). H2A.X: F - ACGAGGAGCTCAACAAGCTG, R - GTGGCGCTG GTCTTCTTG; H2A.J: F - GCTTTTGAATGTGCTGG ATG, R - ACCTCCCCGCTAGATGTCAC; H2A type 3: F - CGGTTGCCGTTGTCTTTTT, R - GAGCGCG ACTTAGCCTTG; H2A type 1C: F - TTAGAGTACC TGACCGCCGA, R - GCGAGTCTTCTTGTTGTCGC; Macro-H2A.1: F - AGACGTCCAGGTCTGCCA, R - CCAGAATCTCCGCTGTCAGG; H2A.V: F - CTACA GTTTCCTGTGGGCCG, R - TCCAGAATCGCAGCA CTGTA; H2A.Z: F - CAGGGAAGGCGGGAGACAG, R - CCTTACCGCCAGCCATTTCG.

### Trypsin digestion

The samples were thawed on ice and processed as previously described [35]. Experiments using a concatenated, heavy-labeled synthetic protein standard demonstrated consistent digestion [51]. The samples were normalized to total protein concentration.

### SRM assay development

SRM assays were developed on a Thermo Scientific TSQ Vantage triple quadrupole mass spectrometer, Accela MS pump, CTC autosampler, and IonMax source equipped with a low-flow metal needle, as previously described [53, 54]. The samples were analyzed in triplicate. Reversed-phase separations were carried out on a 150 × 1-mm Thermo Scientific Hypersil Gold 3.0-μm C18-particle column. Solvent A was LC-MS grade water with 0.2% (v/v) formic acid. Solvent B was LC-MS grade acetonitrile with 0.2% (v/v) formic acid. Thermo Scientific Pinpoint software was used to predict candidate peptides, select fragment ions, and build the MS instrument method.

### Light- and Heavy-labeled peptides

Synthetic isotopically labeled peptides were used as internal standards for quantification. Light and heavy versions (98% purity) of each target peptide were synthesized (Thermo Fisher Scientific). Heavy peptides had sequences identical to those of the light peptides, but the C-terminal Lysine or Arginine was fully-labeled (>98.5%) with 13C or 15N (see Supporting Information Table S1 for peptide sequences and transitions). The peptides were identified by coeluting light and heavy transitions.

### Calibration curve generation

Seven-point calibration curves (0.5, 1, 5, 10, 50, 100, and 500 fmol, Figure 2 a-i) were developed with a pool (30 μg) of all samples as a background matrix. Each point on the calibration curve (and every sample analyzed) included 100 fmol of heavy-labeled peptides. We used reverse curves to generate calibration curves, because we did not have the matrix free of endogenous analytes. Complete details of this type of curve have been presentedpreviously [81]. In addition, all the samples were dissolved in a solution of 200 μg/ml of glucagon in 97% water, 3% ACN, and 0.2% FA to minimize binding to plastic surfaces [54]. Finally, the calibration curve replicate points were run adjacently. As each calibration point contained the same amount of light peptide (endogenous) variance in the heavy peptide (or endogenous peptide), these quantities were used to calculate the run-to-run variance. Also, heavy peptides were spiked into each clinical sample and the low run-to-run variance added confidence to the analysis.

## SUPPORTING DATA, FIGURES AND TABLES


