# Supplementary material for: Depletion of nuclear histone H2A variants is associated with chronic DNA damage signaling upon drug-evoked senescence of human somatic cells
Source: Aging (Albany NY). 2012 Dec 6;4(11):823–42. doi: 10.18632/aging.100507 (PMC3560435; doi:10.18632/aging.100507)
Supplement: Supplementary file 1 [file aging-04-823-s001.pdf]

78. Jasencakova Z, Scharf AN, Ask K, Corpet A, Imhof A, Almouzni G and Groth A. Replication stress interferes with histone recycling and predeposition marking of new histones. Molecular cell. 2010; 37:736-743.

79. Bond JA, Webley K, Wyllie FS, Jones CJ, Craig A, Hupp T and Wynford-Thomas D. p53-Dependent growth arrest and altered p53-immunoreactivity following metabolic labelling with 32P ortho-phosphate in human fibroblasts. Oncogene. 1999; 18:3788-3792.

80. Graham JM. Isolation of nuclei and nuclear membranes from animal tissues. Current protocols in cell biology / editorial board, Juan S Bonifacino et al. 2001; Chapter 3:Unit 3 10.

81. Campbell J, Rezai T, Prakash A, Krastins B, Dayon L, Ward M, Robinson S and Lopez M. Evaluation of absolute peptide quantitation strategies using selected reaction monitoring. Proteomics. 2011; 11:1148-1152.

82. Pospelova TV, Demidenko ZN, Bukreeva EI, Pospelov VA, Gudkov AV, Blagosklonny MV. Pseudo-DNA damage response in senescent cells. Cell Cycle. 2009; 8:4112-4118.

83. Leontieva OV, Lenzo F, Demidenko ZN, Blagosklonny MV. Hyper-mitogenic drive coexists with mitotic incompetence in senescent cells. Cell Cycle. 2012;11: Epub ahead of print.

SUPPORTING DATA

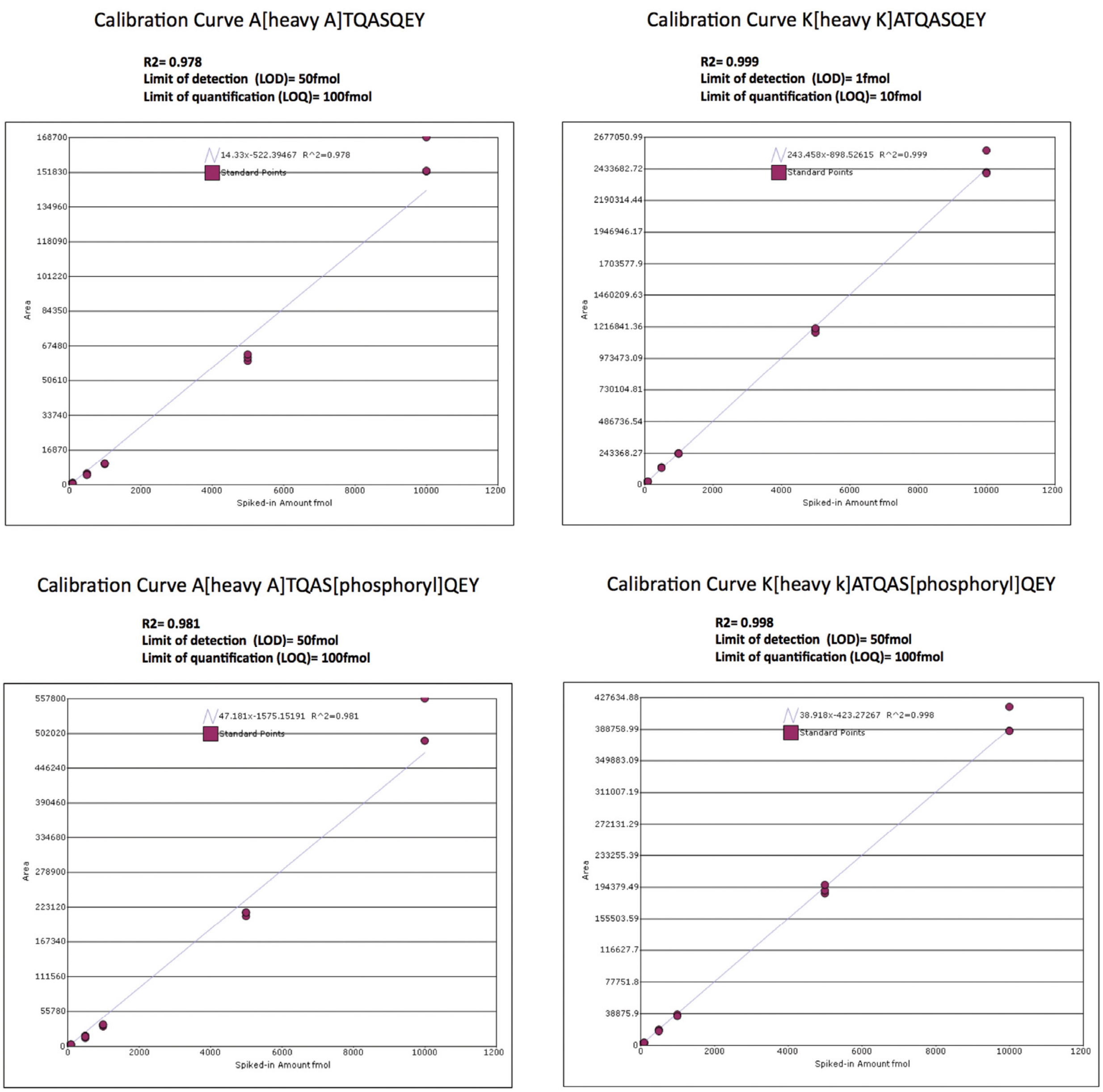

Supporting Figure S1. Peptide calibration curves for SRM analysis.

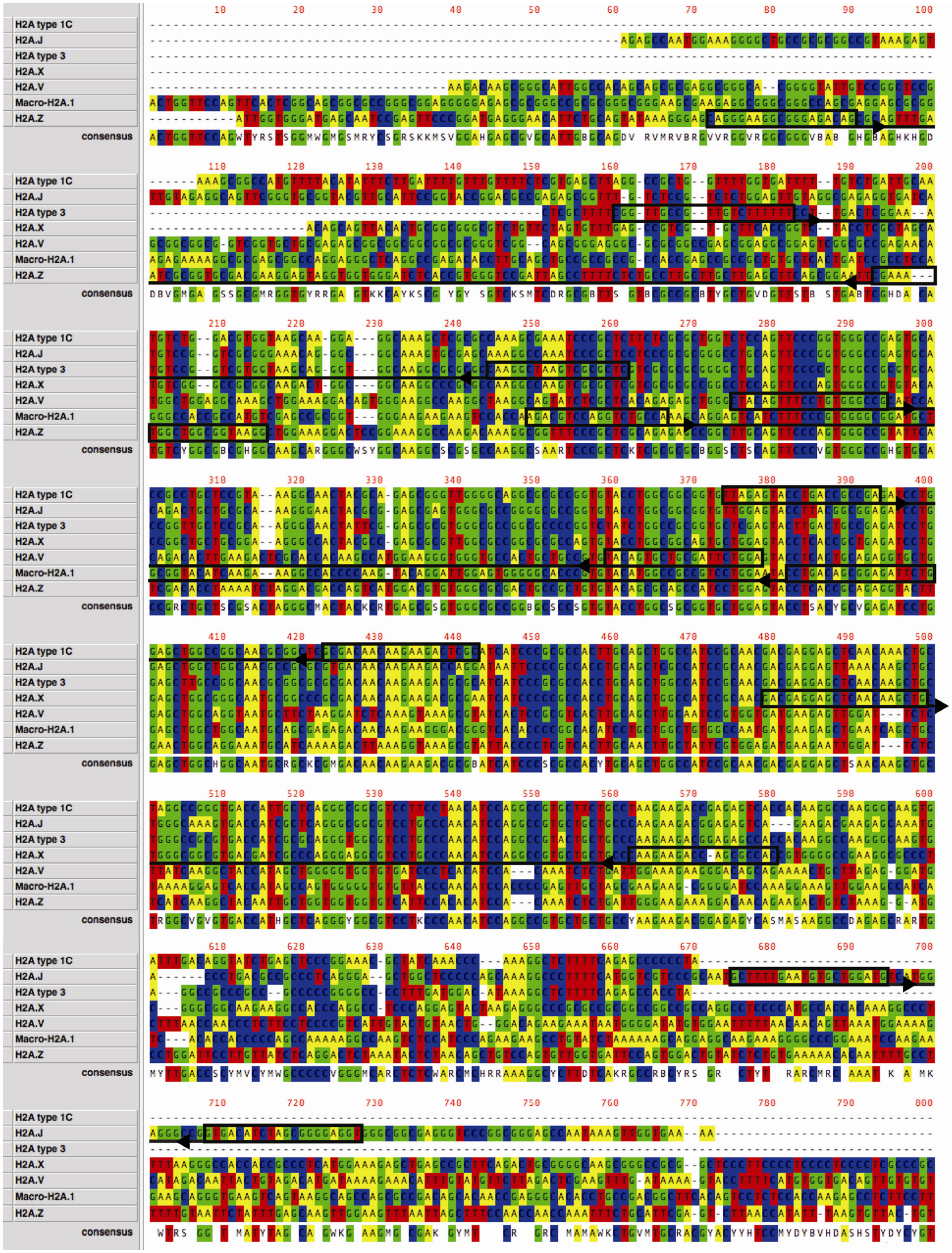

Supporting Figure S2. Genomic sequence ClustalW alignment of a number of histone H2A family members, showing location of qPCR primers. For each H2A variant, the forward and reverse primers for qPCR analysis (designed by BLAST) are highlighted by black boxes. Each resulting amplicon is underlined. The primers for *H2A type 1C* are specific; however, given the high levels of conservation among *H2A type 1* cluster members, all *H2A type 1* variants can be recognized with 2–4 base mismatches, and are also likely to be amplified by these primers. All other primers are specific (>8 mismatches to closest non-specific sequence).

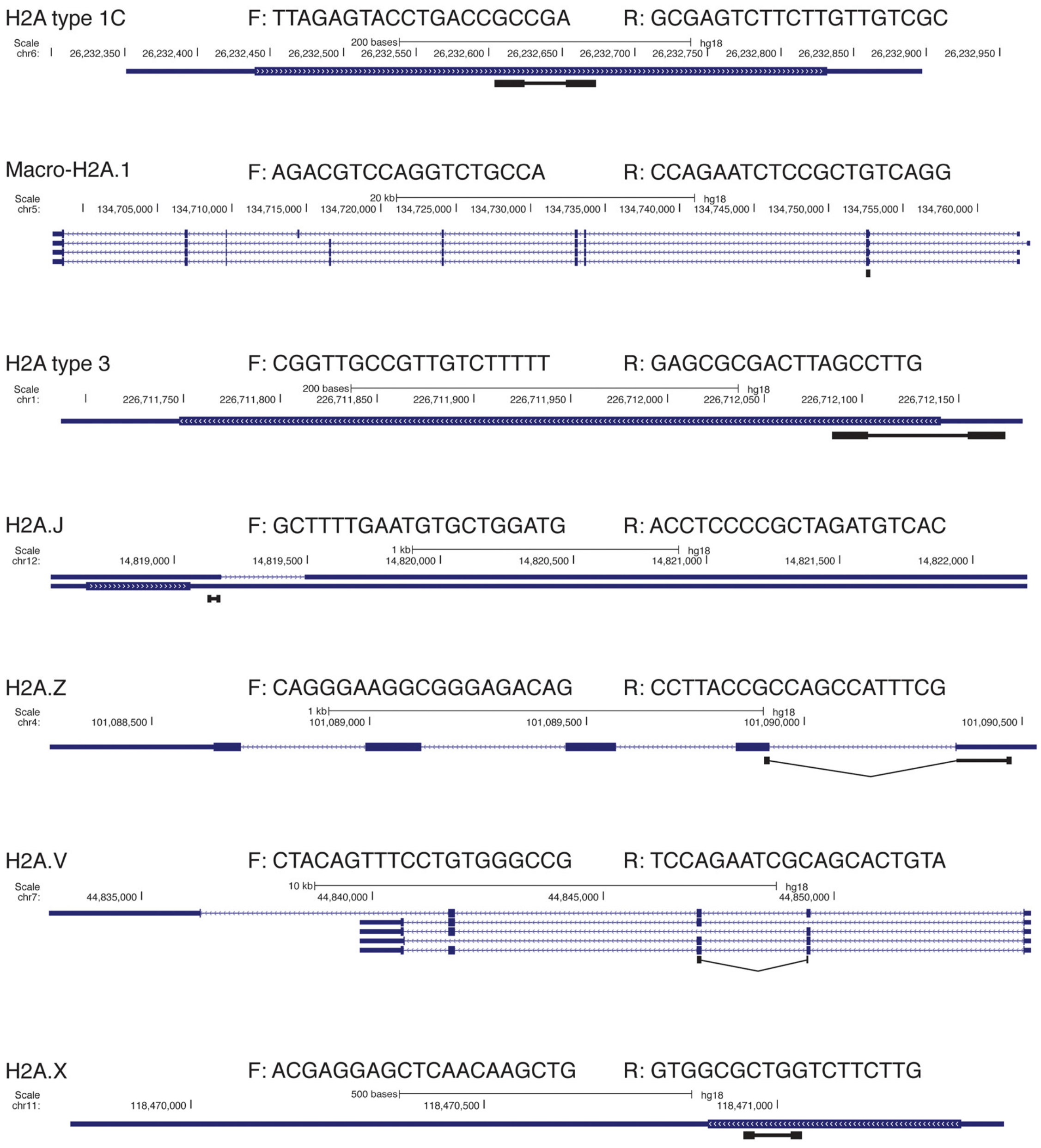

Supporting Figure S3. Location of qPCR primers within each H2A variant genomic sequence.

Table S1. Precursor and product ion masses for target H2A.X peptides.

Precursor m/z	Product m/z	Peptide Sequence	Protein Description	Precursor Charge State	Product Charge State	Ion type
380.713	301.1865	TSATVGPK	Histone H2AX human common to all H2A)	2	1	y3
380.713	400.2549	TSATVGPK	Histone H2AX human common to all H2A)	2	1	y4
380.713	501.3026	TSATVGPK	Histone H2AX human common to all H2A)	2	1	y5
380.713	572.3397	TSATVGPK	Histone H2AX human common to all H2A)	2	1	y6
384.7207	309.2007	TSATVGPK[HeavyK]	Histone H2AX human common to all H2A)	2	1	y3
384.7207	408.2691	TSATVGPK[HeavyK]	Histone H2AX human common to all H2A)	2	1	y4
384.7207	509.3168	TSATVGPK[HeavyK]	Histone H2AX human common to all H2A)	2	1	y5
384.7207	580.3539	TSATVGPK[HeavyK]	Histone H2AX human common to all H2A)	2	1	y6
513.248	439.1818	KATQASQEY	Histone H2AX human unique	2	1	y3
513.248	526.2138	KATQASQEY	Histone H2AX human unique	2	1	y4
513.248	597.2509	KATQASQEY	Histone H2AX human unique	2	1	y5
513.248	725.3095	KATQASQEY	Histone H2AX human unique	2	1	y6
513.248	826.3572	KATQASQEY	Histone H2AX human unique	2	1	y7
553.231	439.1818	KATQAS[Phosphoryl]QEY	Histone H2AX human unique	2	1	y3
553.231	606.1802	KATQAS[Phosphoryl]QEY	Histone H2AX human unique	2	1	y4
553.231	677.2173	KATQAS[Phosphoryl]QEY	Histone H2AX human unique	2	1	y5
553.231	805.2758	KATQAS[Phosphoryl]QEY	Histone H2AX human unique	2	1	y6
553.231	906.3235	KATQAS[Phosphoryl]QEY	Histone H2AX human unique	2	1	y7
557.2388	439.1818	K[HeavyK]ATQAS[Phosphoryl]QEY	Histone H2AX human unique	2	1	y3
557.2388	606.1802	K[HeavyK]ATQAS[Phosphoryl]QEY	Histone H2AX human unique	2	1	y4
557.2388	677.2173	K[HeavyK]ATQAS[Phosphoryl]QEY	Histone H2AX human unique	2	1	y5
557.2388	805.2758	K[HeavyK]ATQAS[Phosphoryl]QEY	Histone H2AX human unique	2	1	y6
557.2388	906.3235	K[HeavyK]ATQAS[Phosphoryl]QEY	Histone H2AX human unique	2	1	y7
517.2556	439.1818	K[HeavyK]ATQASQEY	Histone H2AX human unique	2	1	y3
517.2556	526.2138	K[HeavyK]ATQASQEY	Histone H2AX human unique	2	1	y4
517.2556	597.2509	K[HeavyK]ATQASQEY	Histone H2AX human unique	2	1	y5
517.2556	725.3095	K[HeavyK]ATQASQEY	Histone H2AX human unique	2	1	y6
517.2556	826.3572	K[HeavyK]ATQASQEY	Histone H2AX human unique	2	1	y7
449.201	439.1818	ATQASQEY	Histone H2AX human unique	2	1	y3
449.201	526.2138	ATQASQEY	Histone H2AX human unique	2	1	y4
449.201	597.2509	ATQASQEY	Histone H2AX human unique	2	1	y5
449.201	725.3095	ATQASQEY	Histone H2AX human unique	2	1	y6
489.184	439.1818	ATQAS[Phosphoryl]QEY	Histone H2AX human unique	2	1	y3
489.184	606.1802	ATQAS[Phosphoryl]QEY	Histone H2AX human unique	2	1	y4
489.184	677.2173	ATQAS[Phosphoryl]QEY	Histone H2AX human unique	2	1	y5
489.184	805.2758	ATQAS[Phosphoryl]QEY	Histone H2AX human unique	2	1	y6
491.1877	439.1818	A[HeavyA]TQAS[Phosphoryl]QEY	Histone H2AX human unique	2	1	y3
491.1877	606.1802	A[HeavyA]TQAS[Phosphoryl]QEY	Histone H2AX human unique	2	1	y4
491.1877	677.2173	A[HeavyA]TQAS[Phosphoryl]QEY	Histone H2AX human unique	2	1	y5
491.1877	805.2758	A[HeavyA]TQAS[Phosphoryl]QEY	Histone H2AX human unique	2	1	y6
451.2046	439.1818	A[HeavyA]TQASQEY	Histone H2AX human unique	2	1	y3
451.2046	526.2138	A[HeavyA]TQASQEY	Histone H2AX human unique	2	1	y4
451.2046	597.2509	A[HeavyA]TQASQEY	Histone H2AX human unique	2	1	y5
451.2046	725.3095	A[HeavyA]TQASQEY	Histone H2AX human unique	2	1	y6
472.769	331.2083	AGLQFPVGR	Histone H2AX human common to all H2A)	2	1	y3
472.769	428.261	AGLQFPVGR	Histone H2AX human common to all H2A)	2	1	y4
472.769	575.3295	AGLQFPVGR	Histone H2AX human common to all H2A)	2	1	y5
472.769	703.3881	AGLQFPVGR	Histone H2AX human common to all H2A)	2	1	y6
472.769	816.4721	AGLQFPVGR	Histone H2AX human common to all H2A)	2	1	y7
477.7733	341.2166	AGLQFPVGR[HeavyR]	Histone H2AX human common to all H2A)	2	1	y3
477.7733	438.2693	AGLQFPVGR[HeavyR]	Histone H2AX human common to all H2A)	2	1	y4
477.7733	585.3377	AGLQFPVGR[HeavyR]	Histone H2AX human common to all H2A)	2	1	y5
477.7733	713.3963	AGLQFPVGR[HeavyR]	Histone H2AX human common to all H2A)	2	1	y6
477.7733	826.4803	AGLQFPVGR[HeavyR]	Histone H2AX human common to all H2A)	2	1	y7
425.766	359.2396	HLQLAIR	Histone H2AX human common to all H2A)	2	1	y3
425.766	472.3236	HLQLAIR	Histone H2AX human common to all H2A)	2	1	y4
425.766	600.3822	HLQLAIR	Histone H2AX human common to all H2A)	2	1	y5
430.7706	369.2479	HLQLAIR[HeavyR]	Histone H2AX human common to all H2A)	2	1	y3
430.7706	482.3319	HLQLAIR[HeavyR]	Histone H2AX human common to all H2A)	2	1	y4
430.7706	610.3905	HLQLAIR[HeavyR]	Histone H2AX human common to all H2A)	2	1	y5

Table S2. Abundance ratios and calculated amounts for targeted H2A.X peptides

Peptide sequence	Peptide type	Description	Parent Mass	Ratio of Sample Groups (Group1 : Group2 : Group3)	Internal Standard mass	Proliferating (-Bleo)		Acute DNA Damgae (+Bleo 3hrs)		Drug-evoked Senescence (+Bleo 5days	
						Mean Calculated amount (fmol)	SD	Mean Calculated amount (fmol)	SD	Mean Calculated amount (fmol)	
AGLQFPVGR	Wild type	All H2A variant monitoring peptide	472.77	1.0 : 1.4 : 0.46	517.26	434.03	4160.925818	628.40	3706.169127	200.10	2034.122186
HLQLAIR	Wild type	All H2A variant monitoring peptide	425.77	1.0 : 1.4 : 0.55	517.26	441.71	1036.11349	625.95	738.4782008	243.05	610.8423799
TSATVGPK	Wild type	H2A.X specific monitoring peptide	380.71	1.0 : 1.4 : 0.74	517.26	429.70	15.36752676	598.32	11.54619913	317.18	7.078998618
ATQASQEY	Wild type	C-term serine, fully tryptic	897.39	1.0 : 0.8 : 0.51	901.40	452.10	14.80967227	360.00	16.90857686	230.40	13.68031302
ATQAS*QEY	Phosphorylated	C-term serine, fully tryptic	489.18	1.0 : 4.0 : 2.3	491.19	1.40	0.394968353	5.60	0.422948832	3.28	0.733348485
KATQASQEY	Wild type	C-term serine, missed cleavage	513.25	1.0 : 0.87 : 0.48	517.26	49.39	3.819536013	42.93	2.484851246	23.45	1.629066341
KATQAS*QEY	Phosphorylated	C-term serine, missed cleavage	553.23	1.0 : 2.4 : 1.3	557.24	1.78	0.460310454	4.71	0.854065906	2.24	0.605781667

Peptide sequence	Proliferating (- Bleo)								CV% (Group1)
	Group 1-1 Calculated Amt fmol (05.RAW)	Group 1-2 Calculated Amt fmol (06.RAW)	Group 1-3 Calculated Amt fmol (07.RAW)	Group 1-4 Calculated Amt fmol(08.RAW)	Group 1-5 Calculated Amt fmol (09.RAW)	Group 1-6Calculated Amt fmol (10.RAW)	Group 1-7 Calculated Amt fmol (11.RAW)	Group 1-8 Calculated Amt fmol (12.RAW)	
AGLQFPVGR	95065.56	97262.52	100997.35	106819.29	101687.82	102503.84	100479.50	107086.07	8.00
HLQLAIR	22707.14	23225.00	24276.40	25453.34	24182.24	24511.79	24088.09	25845.65	8.00
TSATVGPK	205.73	238.21	232.41	241.04	223.93	244.65	229.90	257.51	11.00
ATQASQEY	191.82	178.45	212.20	220.34	219.21	205.58	192.55	209.95	15.00
ATQAS*QEY	1.04	0.57	1.37	0.01	0.77	0.87	0.58	0.75	49.00
KATQASQEY	24.06	25.47	29.61	31.56	30.00	21.31	29.71	23.13	11.00
KATQAS*QEY	1.04	0.64	0.51	1.69	0.50	1.20	1.42	0.56	45.00

Group 1= HCA2 proliferating
Group 2= HCA2 3hrs Bleomycin
Group 3= HCA2 3hrs Bleomycin + 5 days

Peptide sequence	Acute DNA damage (+ Bleo 3 hrs)								CV% (Group2)
	Group 2-1 Calculated Amt fmol (17.RAW)	Group 2-2 Calculated Amt fmol (18.RAW)	Group 2-3 Calculated Amt fmol (19.RAW)	Group 2-4 Calculated Amt fmol (20.RAW)	Group 2-5 Calculated Amt fmol (21.RAW)	Group 2-6 Calculated Amt fmol (22.RAW)	Group 2-7 Calculated Amt fmol (23.RAW)	Group 2-8 Calculated Amt fmol (24.RAW)	
AGLQFPVGR	142300.18	140197.38	142959.27	141452.78	142708.19	134281.28	140009.07	133622.19	3.00
HLQLAIR	32719.00	32279.61	33409.47	32891.62	33315.32	31416.52	33550.70	32106.99	4.00
TSATVGPK	302.87	306.32	312.13	304.59	324.05	313.85	327.19	291.88	5.00
ATQASQEY	203.33	169.32	169.32	192.94	201.61	187.32	196.71	218.61	7.00
ATQAS*QEY	3.12	3.07	2.77	2.40	3.05	3.20	3.68	2.43	16.00
KATQASQEY	22.79	17.78	23.16	21.20	22.82	25.88	23.22	19.63	11.00
KATQAS*QEY	1.41	2.22	3.61	2.78	3.19	1.61	1.33	1.89	38.00

Peptide sequence	Drug-evoked senescence (+Bleo 5 days)								CV% (Group3)
	Group 3-1 Calculated Amt fmol (29.RAW)	Group 3-2 Calculated Amt fmol (30.RAW)	Group 3-3 Calculated Amt fmol (31.RAW)	Group 3-4 Calculated Amt fmol (32.RAW)	Group 3-5 Calculated Amt fmol (33.RAW)	Group 3-6 Calculated Amt fmol (34.RAW)	Group 3-7 Calculated Amt fmol (35.RAW)	Group 3-8 Calculated Amt fmol (36.RAW)	
AGLQFPVGR	40031.73	40204.35	39686.50	39419.72	37144.30	35841.82	34931.65	37395.38	3.00
HLQLAIR	11469.70	11527.76	11345.72	11246.86	10652.11	10112.29	9960.07	10801.19	3.00
TSATVGPK	151.87	144.56	148.44	136.10	132.01	135.94	135.47	142.91	5.00
ATQASQEY	114.14	136.44	104.81	119.03	130.02	116.12	109.51	93.23	13.00
ATQAS*QEY	1.64	2.57	1.04	0.39	1.98	1.53	1.08	0.53	51.00
KATQASQEY	12.29	11.53	9.76	8.75	10.17	11.51	7.41	11.06	16.00
KATQAS*QEY	1.00	1.92	1.49	0.68	0.74	1.48	0.11	0.46	54.00
